# Incidence and predictors of root resorption after delayed replantation of permanent teeth

**DOI:** 10.2340/aos.v85.46816

**Published:** 2026-09-23

**Authors:** Yingrui Zhou, Hongwei Ma, Yan Zhang, Haoliang Guo, Ran Ma

**Affiliations:** a Department of General Dentistry, Tianjin Stomatological Hospital, School of Medicine, Nankai University, Tianjin, China; b Dental Emergency Department, Tianjin Stomatological Hospital, School of Medicine, Nankai University, Tianjin, China

**Keywords:** Root resorption, delayed replantation, permanent tooth avulsion, risk factor

## Abstract

Root resorption is a common but unpredictable complication after delayed replantation of avulsed permanent teeth. Evidence from real-world settings regarding its long-term incidence and associated clinical factors remains limited. This study aimed to determine the incidence of root resorption and identify clinical factors associated with its occurrence following delayed replantation of permanent teeth in a real-world tertiary care setting. This retrospective cohort study analyzed patients aged 7–30 years who underwent delayed replantation (> 60 min extra-alveolar time) between 2019 and 2022 at a tertiary dental trauma center, with ≥ 12 months of follow-up. Clinical and radiographic data were reviewed, and root resorption was classified according to predefined radiographic and clinical criteria. Multivariable logistic regression was used to identify factors associated with the occurrence of root resorption, while Kaplan–Meier survival analysis and Cox proportional hazards regression were performed to evaluate the time to first root resorption event. Competing-risk analysis was conducted, considering tooth extraction as a competing event. Among 120 eligible patients (mean age 14 ± 5 years), root resorption occurred in 72 teeth (60.0%), including inflammatory (41.7%) and replacement (58.3%) types based on radiographic and clinical classification. Among all delayed replanted teeth, the estimated tooth survival and functional retention rates at 24 months were 78.3% and 68.3%, respectively. Teeth with root resorption showed longer extra-alveolar times, more frequent dry storage, and delayed or absent root canal treatment (all *P* < 0.01). Multivariable logistic regression identified prolonged extra-alveolar time (> 2 h), dry storage, absence of root canal treatment, and delayed endodontic intervention (> 14 days) as factors associated with a higher occurrence of root resorption. Multivariable logistic regression identified prolonged extra-alveolar time (> 2 h), dry storage, absence of root canal treatment, and delayed endodontic intervention (> 14 days) as factors independently associated with a higher occurrence of root resorption. Root resorption occurred in 60% of delayed replanted teeth in this cohort. Prolonged extra-alveolar time, dry storage, and delayed or absent endodontic treatment were independently associated with a higher occurrence of root resorption. These findings highlight the importance of timely management and appropriate endodontic intervention after delayed replantation; however, the retrospective single-center design limits causal interpretation, and further prospective studies are needed to validate these associations.

## Introduction

Tooth avulsion represents one of the most severe forms of dental trauma and remains a significant clinical challenge in endodontic and trauma management [1]. Replantation of the avulsed permanent tooth is generally regarded as the most biologically favorable treatment option, aiming to restore both esthetics and function [1]. However, despite advances in emergency management and endodontic techniques, the long-term prognosis of replanted teeth remains unpredictable. Among various post-replantation complications, root resorption is considered the most clinically significant cause of treatment failure, as progressive resorption may be associated with tooth mobility, ankylosis, and eventual tooth loss [2–4].

The pathogenesis of post-replantation root resorption is multifactorial, involving mechanical trauma to the periodontal ligament, bacterial contamination, and delayed pulpal or periodontal healing [5–8]. Previous clinical and experimental studies have suggested that extra-oral dry time, storage conditions, root maturity, and tooth type, as well as the timing of endodontic intervention, may influence the development of root resorption [9, 10]. However, reported rates of root resorption vary considerably among studies, ranging from 10% to more than 70%, reflecting substantial differences in patient populations, follow-up periods, treatment protocols, and diagnostic criteria. Furthermore, much of the existing evidence originates from experimental models or small clinical studies performed under controlled conditions, which may not fully represent the complex circumstances encountered in routine clinical practice, where treatment delays and variations in emergency management are common [11].

Delayed replantation, defined as replantation performed after an extra-alveolar time exceeding 60 min, is frequently encountered in daily practice, particularly among children and adolescents who experience dental avulsion in outdoor or sports-related settings [12, 13]. This condition is frequently encountered in children and adolescents because avulsion injuries often occur during outdoor activities or sports, when immediate access to dental care may be limited. Prolonged extra-alveolar exposure, especially under inappropriate storage conditions, has been reported to adversely affect periodontal ligament healing potential and may be associated with a higher likelihood of subsequent root resorption. Although current guidelines recommend replantation whenever feasible, the long-term prognosis of delayed replanted teeth remains variable, and the specific clinical factors influencing treatment outcomes remain incompletely understood.

Therefore, further clinical evidence from real-world patient populations is required to clarify the incidence of root resorption after delayed replantation and to determine the relative contribution of potentially modifiable clinical factors. In particular, understanding the influence of extra-oral time, storage conditions, and timing of endodontic intervention may help identify factors associated with improved clinical management and prognosis. Accordingly, this study aimed to determine the incidence and identify factors independently associated with root resorption following delayed replantation of permanent teeth in a real-world tertiary care setting. Using a consecutive cohort of patients treated between 2019 and 2022, with standardized clinical and radiographic follow-up, we investigated the association between key treatment-related factors and post-replantation outcomes. The findings may contribute to a better understanding of clinical factors associated with root resorption after delayed replantation and provide evidence to support future research and clinical decision-making.

## Methods

### Study design and setting

This investigation was conceived as a retrospective real-world cohort study conducted at the Tianjin Stomatological Hospital, a national tertiary dental referral center in China. The institutional trauma database was systematically reviewed to identify patients presenting with dental avulsion who subsequently underwent replantation between January 2019 and December 2022. All available clinical records, radiographic images, and procedural documentation were reviewed and verified by two independent investigators to ensure data completeness and accuracy, and root resorption, including surface, inflammatory, and replacement types, was evaluated based on radiographic and clinical classification. The primary objective of this study was to determine the incidence and identify clinical factors associated with root resorption following delayed replantation of permanent teeth in routine clinical practice. Patient-, tooth-, and treatment-related variables, including root maturity, tooth type, extra-oral time, storage conditions, and timing of endodontic treatment, were evaluated as potential explanatory variables associated with the observed outcomes.

The reporting of this retrospective cohort study followed the Strengthening the Reporting of Observational Studies in Epidemiology (STROBE) guidelines. A consecutive case identification strategy and standardized inclusion criteria were applied to improve consistency in case selection and reduce potential selection bias. Data collection and follow-up evaluations were completed by December 2024.

### Patient selection and inclusion criteria

Patients were consecutively identified from the institutional registry encompassing all cases of traumatic dental avulsion managed within the specified study period. Eligibility was restricted to individuals aged 7–30 years who had sustained avulsion of permanent teeth subsequently subjected to delayed replantation, defined as extra-alveolar dry time exceeding 60 min.

Patients were included if they had experienced traumatic avulsion of a permanent tooth treated by delayed replantation, had complete baseline clinical information and follow-up radiographic records, and had a minimum follow-up period of 12 months. Patients with follow-up periods extending beyond 24 months were also included when available. Cases were excluded if they involved primary teeth, concomitant root fractures, advanced alveolar bone loss exceeding one-third of the root length, systemic diseases affecting bone metabolism (such as diabetes mellitus or osteoporosis), incomplete documentation of the replantation procedure, or follow-up periods shorter than 12 months. Root resorption was evaluated using predefined clinical and radiographic criteria and classified as surface resorption, inflammatory resorption, or replacement resorption. Relevant clinical variables, including extra-oral time, storage conditions, timing of root canal treatment, root maturity, tooth type, and use of topical fluoride therapy, were extracted from patient records when available. After screening 218 eligible cases, 120 patients fulfilled the study criteria and were included in the final analytical cohort. To maintain statistical independence, only one replanted tooth per patient was included. In patients with multiple avulsed teeth, the tooth with the longest recorded extra-alveolar time or the most complete clinical and radiographic documentation was selected for analysis. Although this approach allowed patient-level analysis, it may introduce selection bias because teeth with more severe trauma characteristics or more complete documentation may have been preferentially selected.

### Clinical procedures and management protocol

In the present study, clinical management protocols were guided by the 2020 International Association of Dental Traumatology (IADT) guidelines for avulsion management [14] which recommend prompt replantation and standardized endodontic intervention based on root maturity and extra-oral time. However, as this was a retrospective real-world cohort study, variations in clinical decision-making and treatment timing occurred according to individual patient circumstances, injury severity, clinical availability, and clinician judgment. Our work aligns with prior clinical cohorts investigating delayed replantation, which have reported variable tooth survival and root resorption outcomes depending on extra-oral duration, storage medium, and timing of endodontic therapy [15–17]. All replanted teeth were evaluated according to predefined clinical and radiographic criteria for early superficial root surface changes, inflammatory resorption, and replacement resorption. Early superficial root surface changes were recorded as radiographic alterations compatible with superficial root changes during the early healing period; because histological confirmation was unavailable, these findings were not considered definitive diagnoses of microscopic surface resorption. Soft tissue changes, including partial root denudation due to loss of epithelial attachment, were documented. Topical fluoride applications were recorded in teeth exhibiting delayed replacement resorption as part of post-replantation management. Because histopathological evaluation was not feasible in this clinical cohort, all resorption classifications were based on clinical and radiographic criteria, and findings should therefore be interpreted as clinically diagnosed resorption patterns rather than histologically confirmed pathological processes. By incorporating guideline-based procedures and clearly defining clinically measurable outcomes, this study aimed to reflect real-world treatment practices while avoiding overinterpretation of findings that cannot be confirmed histologically.

All clinical interventions were performed by senior endodontists following a standardized management algorithm derived from the 2020 IADT guidelines. Although a guideline-based algorithm was used, individual treatment decisions were not randomized or assigned by protocol and may have been influenced by patient-specific clinical factors. In line with IADT guidelines, delayed replantation procedures (extra-oral time >60 min) were performed following standard protocol whenever feasible. For cases in which the timing or performance of root canal treatment (RCT) differed from the recommended protocol, these variations were systematically recorded and included as treatment-related variables in subsequent statistical analyses to evaluate real-world clinical variability. The definitions, sources of data collection, and categorization strategies for trauma-related, tooth-related, and treatment-related variables included in the statistical analyses are summarized in Supplementary Table S1. To avoid duplication, a single tooth per patient was analyzed, prioritizing either the tooth with the longest extra-oral time or the tooth with the most complete documentation. This approach reduced dependency between observations but may limit generalizability and could introduce selection bias because only one tooth per patient was included. Upon presentation, avulsed teeth were gently cleansed with sterile saline to remove debris and non-vital tissue remnants. The alveolar socket was irrigated to remove coagulum before gentle replantation under local anesthesia. When extra-oral time exceeded 60 min, the root surface was managed according to delayed replantation protocols, including cleansing and saline rehydration before repositioning.

#### Splinting protocol

Flexible splints were routinely applied for 10–14 days using 0.3-mm orthodontic wire or nylon fiber reinforced with composite resin. Rigid splinting was selectively used in cases with severe mobility, associated root fractures, or alveolar bone injury according to clinician judgment. Because splint selection was determined clinically rather than randomly assigned, the association between splinting characteristics and outcomes may be influenced by injury severity and other clinical factors.

#### Extra-oral time definition

Extra-oral time was defined as the total duration from avulsion to tooth replantation. Dry time, defined as the duration during which the tooth remained outside a moist storage environment, was recorded separately when available to distinguish the effect of total extra-oral duration from the specific impact of dehydration-related periodontal ligament injury.

Systemic antibiotics, typically amoxicillin with or without clavulanate, were administered for 5 to 7 days unless contraindicated. Patients were instructed to maintain meticulous oral hygiene and to use chlorhexidine mouth rinse twice daily.

#### Endodontic timing

Root canal therapy (RCT) was performed within 1–2 weeks whenever feasible, according to IADT recommendations. The interval between replantation and initiation of RCT was recorded as an independent treatment-related variable. Delayed endodontic intervention was categorized as >14 days and >4 weeks based on previous literature and guideline recommendations regarding higher occurrence of inflammatory complications. Continuous analysis using exact days to RCT was also performed to evaluate the relationship between treatment timing and resorption outcomes. Calcium hydroxide was used as an intracanal medicament and renewed at 3-month intervals until obturation.

Patients were scheduled for follow-up examinations at 3, 6, 12, 18, and 24 months after replantation. At each visit, standardized clinical and radiographic evaluations were performed to assess periodontal healing, tooth stability, and post-replantation complications, including radiographically detectable root surface changes, inflammatory resorption, replacement resorption, ankylosis, and infection-related changes.

### Radiographic and clinical evaluation

Standardized periapical radiographs were obtained at baseline and at each scheduled follow-up visit using the paralleling technique (Planmeca ProX, Helsinki, Finland). Radiographic images were acquired using consistent exposure parameters to ensure comparability across examinations. When radiographic findings suggested advanced or uncertain pathological changes, cone-beam computed tomography (CBCT) was performed to further evaluate root integrity and surrounding bone structures. Root resorption was assessed using predefined clinical and radiographic criteria rather than histopathological evaluation. Therefore, the diagnosis represented clinically and radiographically detectable resorption rather than direct confirmation of microscopic pathological changes. Two calibrated examiners independently reviewed all radiographs while blinded to clinical information, and disagreements were resolved through consensus discussion. Inter-observer agreement was evaluated using Cohen’s κ coefficient, with a value of 0.86 indicating strong agreement. Although examiner calibration and blinded assessment were applied to improve measurement reliability, radiographic evaluation may underestimate early microscopic changes that are not detectable through conventional imaging techniques.

Clinical examinations included assessment of tooth mobility according to the Miller index, percussion sound evaluation for ankylosis, and periodontal probing at six sites per tooth using a calibrated periodontal probe. The presence of pain, sinus tract formation, gingival inflammation, and other clinical signs associated with post-replantation complications were systematically recorded.

### Definition and classification of root resorption

Root resorption was diagnosed and categorized according to previously established radiographic and clinical criteria recommended in the literature and by the IADT guidelines. The evaluated categories included early superficial root changes, inflammatory resorption, and replacement resorption. Early superficial root changes were recorded when small localized radiographic alterations of the root surface were observed without evidence of progressive inflammatory or replacement resorption. Because histological confirmation was unavailable in this clinical cohort, these findings were considered radiographic features compatible with superficial resorption rather than definitive histological diagnoses of surface resorption.

Inflammatory resorption was diagnosed when radiographs demonstrated irregular radiolucent defects along the root surface accompanied by loss of lamina dura continuity and/or adjacent periradicular radiolucency. Clinical findings, including pain, tenderness, or sinus tract formation, were considered supportive diagnostic features. The diagnosis of inflammatory resorption was therefore based on a combination of radiographic findings and supportive clinical signs when present; the presence of these features indicates clinically apparent inflammatory resorption but does not establish the underlying biological mechanism with absolute certainty. Replacement resorption (ankylosis-related resorption) was identified by progressive loss of the periodontal ligament space, direct contact between bone and root surface, and clinical evidence of ankylosis, such as a metallic percussion sound and reduced physiological mobility. Because replacement resorption was identified using clinical and radiographic indicators, rather than histological examination, the diagnosis reflects ankylosis-related radiographic and clinical changes observed during follow-up.

The onset of root resorption was defined as the earliest follow-up examination at which definitive radiographic or clinical evidence of inflammatory or replacement resorption was identified. For early superficial root changes, the earliest radiographic appearance of localized root surface alterations was recorded. All diagnoses were confirmed by both examiners to reduce observer-related variability.

### Outcome measures

The primary outcome of this study was the occurrence of root resorption following delayed replantation during the 24-month follow-up period. For descriptive analyses and binary regression modeling, root resorption was evaluated as a dichotomous outcome (presence or absence). Multivariable logistic regression was used to identify factors associated with the occurrence of root resorption.

Secondary outcomes included time-dependent resorption outcomes, specifically time to inflammatory resorption and time to replacement resorption. For these outcomes, the time origin was defined as the date of tooth replantation, and event time was calculated as the interval between replantation and the first documented occurrence of the corresponding resorption event. The event date was defined as the earliest follow-up examination showing definitive clinical and/or radiographic evidence of inflammatory resorption or replacement resorption according to predefined diagnostic criteria. These time-dependent outcomes were analyzed using Kaplan–Meier survival analysis and Cox proportional hazards regression models.

Additional secondary outcomes included overall tooth survival, defined as the interval from replantation to extraction; functional retention, defined as maintenance of an asymptomatic tooth in functional occlusion; periodontal parameters including probing depth and tooth mobility; and post-replantation complications including ankylosis and infection-related events.

Outcome data were obtained from longitudinal clinical records and standardized radiographic and clinical assessments performed during scheduled follow-up examinations. Patients who did not experience the specific resorption event during the observation period were censored at their last available follow-up visit or at 24 months, whichever occurred first. When tooth extraction occurred before documentation of the analyzed resorption event, extraction was considered a competing event because it prevented further observation of the specific resorption outcome, and competing-risk analysis was performed using the Fine–Gray subdistribution hazard model.

Root resorption outcomes were diagnosed and categorized according to predefined clinical and radiographic criteria rather than histopathological evaluation. Inflammatory resorption was defined by irregular radiolucent defects along the root surface, disruption of lamina dura continuity, and/or associated periradicular radiolucency with supportive clinical findings when present. Replacement resorption (ankylosis-related resorption) was characterized by progressive loss of the periodontal ligament space, direct bone-to-root contact, and clinical evidence of ankylosis, including metallic percussion sound and reduced physiological mobility.

The variables included in each statistical model were prespecified based on clinical relevance, previous literature, and guideline recommendations. These variables included patient-related factors (age and sex), tooth-related factors (root maturity and tooth type), trauma-related factors (extra-alveolar time and storage condition), and treatment-related factors (root canal treatment status and timing, splinting protocol, and initial tooth mobility). The relationship between outcomes, statistical methods, and included variables is summarized in Supplementary Table S1.

All endpoint assessments were performed according to predefined diagnostic thresholds to minimize information bias and improve consistency between evaluators. This assessment framework allowed evaluation of both the occurrence and timing of resorption events and their association with tooth survival and functional outcomes.

### Ethical approval

The research protocol was reviewed and approved by the Institutional Ethics Committee of Tianjin Stomatological Hospital (approval number TSH202508193). The study was conducted in accordance with the principles of the Declaration of Helsinki (2013 revision) and relevant national regulations governing biomedical research involving human subjects. Because of the retrospective nature of the study and the use of previously collected clinical data, the requirement for informed consent was waived by the ethics committee. All patient-related information was anonymized before data extraction, and each case was assigned a unique identification code to ensure confidentiality.

All extracted data were stored on secure institutional servers with restricted access limited to authorized investigators. The funding sources, if any, did not influence study design, data analysis, interpretation of results, or manuscript preparation.

### Statistical analysis

All statistical analyses were conducted at the patient level, with one replanted tooth per patient included in the final dataset to ensure independence of observations. The statistical approach was selected according to the type and timing of each outcome. Binary occurrence of root resorption was evaluated using logistic regression analysis, whereas time-to-event outcomes, including time to inflammatory resorption and replacement resorption, were analyzed using Kaplan–Meier survival analysis and Cox proportional hazards regression models. When tooth extraction occurred before documentation of the resorption event, Fine–Gray competing-risk regression was used to account for extraction as a competing event. The variables included in each statistical model were selected based on clinical relevance and are summarized in Supplementary Table S2. Continuous variables were assessed for distributional characteristics using normality testing and were presented as mean ± standard deviation (SD) or median with interquartile range (IQR), as appropriate. Categorical variables were summarized as frequencies and percentages. Baseline comparisons between patients with and without root resorption were performed using the independent-samples *t*-test or Mann–Whitney U test for continuous variables and the χ² test or Fisher’s exact test for categorical variables, according to data distribution and expected cell counts. Longitudinal changes in periodontal parameters during follow-up were evaluated using repeated-measures analysis of variance (ANOVA) when assumptions of normality and sphericity were satisfied. Appropriate post-hoc adjustments were applied for multiple comparisons. For binary outcome analysis, the primary outcome of root resorption was defined as the presence or absence of any clinically or radiographically detected resorption during the 24-month follow-up period. Multivariable logistic regression models were constructed to identify factors independently associated with the occurrence of root resorption. For time-to-event analyses, the time origin was defined as the date of tooth replantation. Event time was calculated as the interval between replantation and the first documented occurrence of the specific resorption event. For inflammatory and replacement resorption, the event date was defined as the earliest follow-up examination demonstrating definitive clinical and/or radiographic evidence according to predefined diagnostic criteria. Patients without documented events were censored at their last available follow-up examination or at 24 months, whichever occurred first. Kaplan–Meier curves were generated to estimate the cumulative incidence of inflammatory and replacement resorption over time, and Cox proportional hazards regression models were used to evaluate factors associated with the timing of occurrence of each resorption outcome.

Variables included in multivariable models were selected based on both clinical relevance and statistical findings from univariate analyses. Variables with *P* < 0.10 in univariate analyses, together with clinically important potential confounders identified from previous literature and clinical guidelines, were considered for multivariable adjustment. The evaluated covariates included patient-related factors (age and sex), tooth-related factors (root maturity and tooth type), trauma-related factors (extra-alveolar time and storage condition), and treatment-related factors (root canal treatment status, timing of root canal treatment, splinting protocol, and initial tooth mobility), as summarized in Supplementary Table S2. For binary outcomes, multivariable logistic regression was performed using a backward likelihood-ratio approach. For time-dependent outcomes, Cox proportional hazards models were constructed with adjustment for clinically relevant covariates. Effect estimates were reported as odds ratios (ORs) or hazard ratios (HRs) with corresponding 95% confidence intervals (CIs). Because tooth extraction may prevent observation of subsequent root resorption, Fine–Gray subdistribution hazard models were applied when appropriate, with extraction treated as a competing event. This approach allowed estimation of the association between clinical variables and replacement resorption while accounting for the possibility that extraction altered the observed occurrence of the resorption outcome.

Model assumptions and diagnostic performance were assessed before interpretation of regression results. The proportional hazards assumption for Cox regression models was evaluated using Schoenfeld residuals. Logistic regression models were assessed using the Hosmer–Lemeshow goodness-of-fit test, and diagnostic evaluation was performed to assess potential model instability.

Sensitivity analyses were performed to evaluate the robustness of the findings. These analyses included assessment of the influence of the single-tooth-per-patient selection strategy on survival outcomes and replacement resorption estimates. All statistical analyses were performed using IBM SPSS Statistics version 26.0 (IBM Corp., Armonk, NY, USA). All statistical tests were two-sided, and a *P*-value < 0.05 was considered statistically significant.

## Results

### Patient characteristics and baseline comparisons

**Figure 1:**
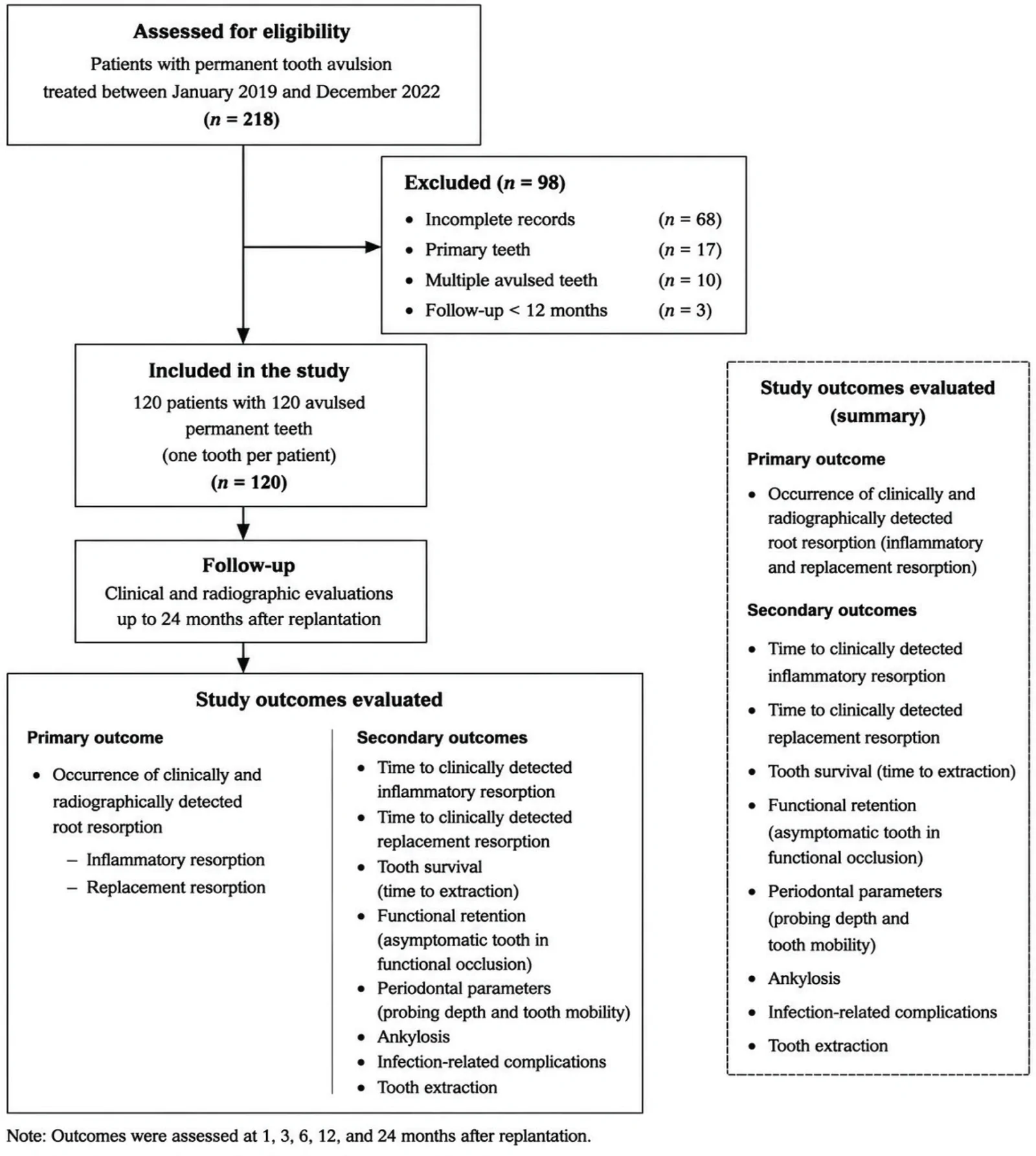
Flowchart of patient selection, follow-up, and study outcomes. A total of 218 patients with avulsed permanent teeth replanted between January 2019 and December 2022 were screened. After exclusions, 120 patients (one replanted tooth per patient) were included in the final analysis. The primary outcome was clinically and radiographically detected root resorption, and secondary outcomes included time to resorption events, tooth survival, functional retention, periodontal parameters, and post-replantation complications.

A total of 218 patients with traumatic tooth avulsion who underwent replantation between January 2019 and December 2022 were retrospectively screened from the institutional dental trauma registry. After excluding cases with incomplete records (*n* = 68), primary teeth (*n* = 17), multiple avulsed teeth (*n* = 10), and loss to follow-up within 12 months (*n* = 3), 120 patients with one replanted permanent tooth per patient were included in the final analysis (Figure 1).

During the 24-month follow-up period, root resorption was detected clinically and radiographically in 72 of 120 replanted teeth (60.0%), whereas 48 teeth (40.0%) showed no evidence of resorption. Among teeth with root resorption, inflammatory resorption and replacement resorption accounted for 41.7% and 58.3% of cases, respectively. Baseline demographic, clinical, and treatment-related characteristics of teeth with and without root resorption are summarized in Table 1.

**Table 1:** Baseline characteristics and clinical variables associated with root resorption after delayed replantation (*n* = 120).

Variable	With root resorption (*n* = 72)	Without root resorption (*n* = 48)	Statistical test (value)	*P*
**Demographics**				
Age (years), mean ± SD	14 ± 5	13 ± 4	*t*-test (*t* = 1.21)	0.23
Male, *n* (%)	58.3% (42/72)	54.2% (26/48)	χ² test (χ² = 0.18)	0.67
Type of trauma (fall/sports/traffic), *n* (%)	84.7% (61/72)	81.3% (39/48)	χ² test (χ² = 0.24)	0.63
**Tooth and injury characteristics**				
Maxillary central incisor, *n* (%)	63.9% (46/72)	58.3% (28/48)	χ² test (χ² = 0.38)	0.54
Mature root (closed apex), *n* (%)	41.7% (30/72)	58.3% (28/48)	χ² test (χ² = 3.84)	*0.05*
Associated crown/root fracture, *n* (%)	15.3% (11/72)	8.3% (4/48)	χ² test (χ² = 1.27)	0.26
Alveolar bone fracture, *n* (%)	18.1% (13/72)	14.6% (7/48)	χ² test (χ² = 0.23)	0.63
**Replantation timing and storage**				
Extra-alveolar time (h), mean ± SD	2.6 ± 1.3	1.6 ± 0.9	*t*-test (*t* = 4.52)	< 0.001*
Delay > 2 h, *n* (%)	77.8% (56/72)	41.7% (20/48)	χ² test (χ² = 13.92)	< 0.001*
Storage condition (dry), *n* (%)	61.1% (44/72)	29.2% (14/48)	χ² test (χ² = 11.56)	0.001*
**Treatment factors**				
Flexible splint fixation, *n* (%)	83.3% (60/72)	85.4% (41/48)	χ² test (χ² = 0.09)	0.77
Splinting duration (days), mean ± SD	13 ± 2	13 ± 2	*t*-test (*t* = 0.28)	0.78
Root canal treatment ≤ 2 weeks, *n* (%)	45.8% (33/72)	81.3% (39/48)	χ² test (χ² = 15.61)	< 0.001*
Delayed or no RCT (> 4 weeks), *n* (%)	43.3% (31/72)	18.8% (9/48)	χ² test (χ² = 7.62)	0.006*
Use of calcium hydroxide, *n* (%)	41.7% (30/72)	56.3% (27/48)	χ² test (χ² = 2.49)	0.12
Use of NaOCl root cleaning, *n* (%)	40.3% (29/72)	29.2% (14/48)	χ² test (χ² = 1.43)	0.23
Antibiotic prescribed, *n* (%)	90.3% (65/72)	91.7% (44/48)	χ² test (χ² = 0.06)	0.81
**Initial and follow-up conditions**				
Tooth mobility ≥ grade II, *n* (%)	30.6% (22/72)	10.4% (5/48)	χ² test (χ² = 6.36)	0.01*
Periodontal pocket depth (mm), mean ± SD	2.6 ± 0.8	2.4 ± 0.7	*t*-test (*t* = 1.38)	0.17
Follow-up duration (months), mean ± SD	20 ± 5	21 ± 5	*t*-test (*t* = 0.93)	0.35

SD: standard deviation; RCT: root canal treatment; NaOCl: sodium hypochlorite.

Note: Continuous variables are expressed as mean ± SD; categorical variables are shown as percentage (*n*/%). Continuous variables were compared using independent-sample *t*-tests, and categorical variables were compared using χ² tests or Fisher’s exact tests, as appropriate.

* *P* < 0.05 indicate statistical significance.

The mean age of the cohort was 14 ± 5 years, and 56.7% of patients were male. No significant differences were observed between the resorption and non-resorption groups regarding age, sex, or trauma etiology (all *P* > 0.05). Several treatment-related factors differed significantly between groups. Teeth that developed root resorption had a longer extra-alveolar time compared with teeth without resorption (2.6 ± 1.3 h vs. 1.6 ± 0.9 h, *P* < 0.001), and delayed replantation exceeding 2 h was more frequent among teeth with root resorption (77.8% vs. 41.7%, *P* < 0.001).

Storage conditions before replantation were also associated with root resorption. Dry storage was more common among teeth with root resorption (61.1% vs. 29.2%, *P* = 0.001), whereas physiological storage media (saline or milk) were more frequently observed among teeth without resorption (22.2% vs. 43.8%, *P* = 0.02). Immature teeth showed a higher proportion of root resorption compared with mature teeth (58.3% vs. 41.7%, *P* = 0.05).

Treatment-related variables also differed between groups. Teeth with root resorption were less likely to receive root canal treatment within 2 weeks after replantation compared with teeth without resorption (45.8% vs. 81.3%, *P* < 0.001), and the interval between replantation and root canal treatment was longer in teeth that developed resorption (13 ± 4 days vs. 9 ± 3 days, *P* < 0.001). Increased initial tooth mobility (≥ grade II) was also more frequently observed among teeth with root resorption (30.6% vs. 10.4%, *P* = 0.01).

Overall, prolonged extra-alveolar time, unfavorable storage conditions, delayed endodontic treatment, and increased initial mobility were associated with the occurrence of root resorption in unadjusted comparisons. These variables were subsequently evaluated in multivariable regression models to determine their independent associations with root resorption.

**Table 4:** Cox proportional hazards regression analysis for time to resorption.

Variable	Adjusted HR	95% CI	*P*
Inflammatory resorption: Extra-alveolar time >2 h	1.74	0.89–3.41	0.104
Inflammatory resorption: Dry storage	1.62	0.84–3.12	0.148
Inflammatory resorption: Absence of RCT	2.05	1.02–4.12	0.044
Inflammatory resorption: Delayed RCT >14 days	1.72	0.89–3.34	0.106
Inflammatory resorption: Immature root	2.31	1.17–4.57	0.016
Replacement resorption: Extra-alveolar time >2 h	2.94	1.38–6.27	0.005
Replacement resorption: Dry storage	2.52	1.18–5.38	0.017
Replacement resorption: Absence of RCT	3.41	1.55–7.50	0.002
Replacement resorption: Delayed RCT >14 days	2.11	1.01–4.41	0.047
Replacement resorption: Mature root	2.04	1.01–4.13	0.046

HR: hazard ratio; CI: confidence interval; RCT: root canal treatment.

Time origin: Date of tooth replantation. Patients without the event were censored at the last available follow-up or at 24 months.

### Characteristics and temporal evolution of root resorption following delayed replantation

Among the 72 replanted permanent teeth that developed root resorption during the 24-month follow-up period, resorption patterns were classified according to predefined clinical and radiographic criteria as inflammatory resorption, replacement resorption, or early superficial root surface changes (Table 2). Early superficial root surface changes were mainly detected during the initial follow-up period (within 3–4 months after replantation) and remained limited without clinically detectable progression during the observation period.

**Table 2:** Comparison of clinical variables associated with inflammatory and replacement root resorption following delayed replantation.

Parameter	Inflammatory (*n* = 30)	Replacement (*n* = 42)	Statistical test (value)	*P*
**Onset time of resorption (months), mean ± SD**	4.6 ± 1.9	15.0 ± 3.7	*t*-test (*t* = 10.9)	< 0.001*
**Tooth location**				
Maxillary central incisor, *n* (%)	63.3% (19/30)	59.5% (25/42)	χ² test (χ² = 0.12)	0.73
Other anterior teeth (lateral/canine), *n* (%)	36.7% (11/30)	40.5% (17/42)		
**Root development status**				
Mature root (closed apex), *n* (%)	30.0% (9/30)	54.8% (23/42)	χ² test (χ² = 4.10)	0.04*
Immature root (open apex), *n* (%)	70.0% (21/30)	45.2% (19/42)		
**Replantation factors**				
Extra-alveolar time > 2 h, *n* (%)	83.3% (25/30)	76.2% (32/42)	χ² test (χ² = 0.49)	0.49
Dry storage before replantation, *n* (%)	70.0% (21/30)	59.5% (25/42)	χ² test (χ² = 0.77)	0.38
**Endodontic management**				
RCT within 2 weeks, *n* (%)	36.7% (11/30)	54.8% (23/42)	χ² test (χ² = 2.29)	0.13
Delayed or no RCT (> 4 weeks), *n* (%)	43.3% (13/30)	19.0% (8/42)	χ² test (χ² = 4.72)	0.03*
Time to RCT completion (days), mean ± SD	14 ± 4	10 ± 3	*t*-test (*t* = 4.13)	<0.001*

SD: standard deviation; RCT: root canal treatment.

Note: Continuous variables are expressed as mean ± SD; categorical variables as percentage (*n*/%). Independent-sample *t*-tests were used for continuous variables, whereas χ² tests or Fisher’s exact tests were used for categorical variables when appropriate. *P* < 0.05 was considered statistically significant.

**Figure 2:**
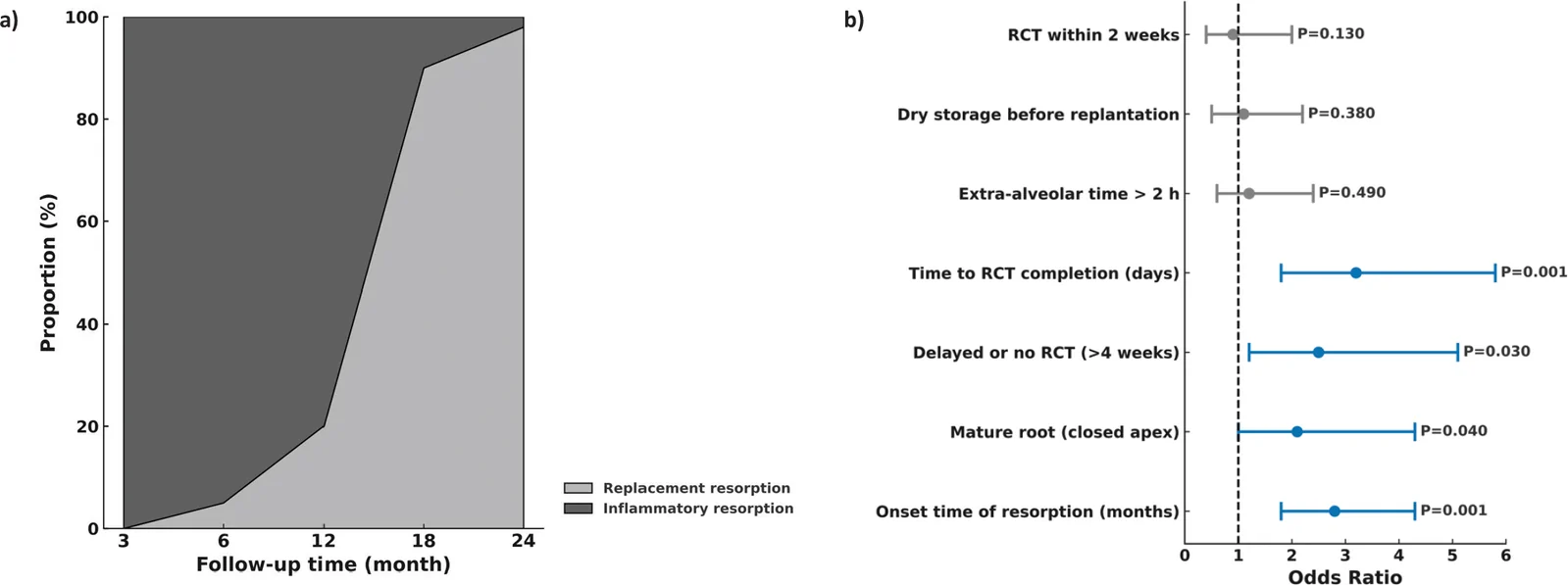
Patterns and clinical characteristics of root resorption following delayed replantation. (A) Temporal distribution of inflammatory and replacement root resorption over the 24-month follow-up period. Time-dependent occurrence of resorption events was evaluated using Kaplan–Meier survival analysis. (B) Comparison of clinical and procedural characteristics between inflammatory and replacement resorption types. Continuous variables were compared using independent-sample *t*-tests, and categorical variables were compared using χ² tests or Fisher’s exact tests, as appropriate. Significant variables (*P* < 0.05) are highlighted in blue, whereas nonsignificant parameters are shown in gray.

Inflammatory and replacement resorption demonstrated different temporal patterns. Inflammatory resorption was detected earlier after replantation, with a mean detection time of 4.6 ± 1.9 months, whereas replacement resorption developed later, with a mean onset time of 15.0 ± 3.7 months (*P* < 0.001). During longitudinal follow-up, inflammatory resorption was predominantly observed during the early months after replantation, while replacement resorption became increasingly evident after 12 months and represented the predominant late resorption pattern at 24 months (Figure 2A).

The distribution of resorption types varied according to root maturity. Replacement resorption occurred more frequently in teeth with mature roots compared with immature roots (54.8% vs. 45.2%, *P* = 0.04), whereas inflammatory resorption was more commonly observed among teeth with immature roots (70.0%). Extra-alveolar time exceeding 2 h was frequent among both inflammatory and replacement resorption groups, with no significant difference between resorption types (83.3% vs. 76.2%, *P* = 0.49).

Overall, root resorption after delayed replantation demonstrated distinct patterns according to resorption type, with inflammatory resorption occurring earlier and replacement resorption developing predominantly during later follow-up.

### Clinical outcomes and complication trends following delayed replantation

**Figure 3:**
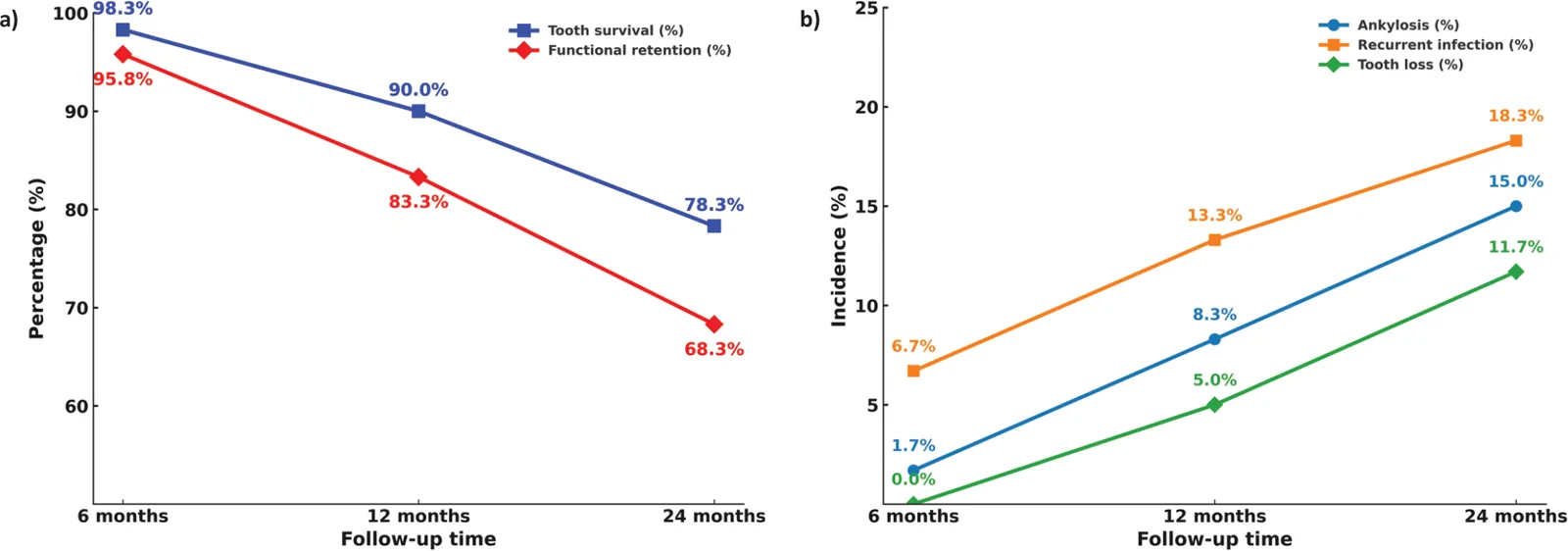
Long-term clinical outcomes and complication trends following delayed tooth replantation. (A) Tooth survival and functional retention rates at 6, 12, and 24 months after delayed replantation. Tooth survival and functional retention were estimated using Kaplan–Meier survival analysis. (B) Incidence of major complications, including ankylosis, recurrent infection, and tooth loss due to severe resorption, at corresponding follow-up intervals. Temporal changes in categorical complications were evaluated using χ² tests for trend. Data are presented as percentages with one decimal place, rounded for consistency with Table 3.

Secondary clinical outcomes during the 24-month follow-up period were evaluated to describe tooth retention and post-replantation complications (Table 3, Figure 3A). The survival and functional retention of replanted teeth gradually declined during follow-up. The cumulative tooth survival rate decreased from 98.3% (118/120) at 6 months to 90.0% (108/120) at 12 months and 78.3% (94/120) at 24 months (Cochran’s *Q* = 19.8, *P* < 0.001). Similarly, functional retention decreased from 95.8% (115/120) at 6 months to 83.3% (100/120) at 12 months and 68.3% (82/120) at 24 months (χ² = 22.6, *P* < 0.001) (Table 3, Figure 3A).

**Table 3:** Secondary complications and periodontal outcomes during 24-month follow-up after delayed replantation.

Clinical outcome	6 months	12 months	24 months	Temporal analysis/P-value
Ankylosis detected, *n* (%)	2 (1.7%, 2/120)	10 (8.3%, 10/120)	18 (15.0%, 18/120)	χ² test for trend (χ² = 8.9); *P* = 0.003*
Recurrent infection (sinus/fistula), *n* (%)	8 (6.7%, 8/120)	16 (13.3%, 16/120)	22 (18.3%, 22/120)	χ² test for trend (χ² = 7.2); *P* = 0.01*
Tooth extraction due to severe resorption, *n* (%)	0 (0.0%, 0/120)	6 (5.0%, 6/120)	14 (11.7%, 14/120)	χ² test for trend (χ² = 10.3); *P* = 0.002*
Mean probing depth (mm), mean ± SD	2.3 ± 0.7	2.5 ± 0.8	2.8 ± 0.9	Repeated-measures ANOVA (*F* = 6.4); *P* = 0.002*
Tooth mobility ≥ grade II, *n* (%)	18 (15.0%, 18/120)	24 (20.0%, 24/120)	28 (23.3%, 28/120)	χ² test for trend (χ² = 3.1); *P* = 0.21

SD: standard deviation; ANOVA: analysis of variance.

Note: Values represent temporal trends during follow-up. Because follow-up completeness varied across time points, analyses describe changes over time rather than paired comparisons among all participants. Continuous variables are presented as mean ± SD, and categorical variables are expressed as *n* (%). Temporal changes in categorical outcomes were evaluated using χ² tests for trend, and longitudinal changes in probing depth were assessed using repeated-measures ANOVA.

* *P* < 0.05 indicates statistical significance.

During follow-up, post-replantation complications increased over time. Mean periodontal probing depth increased from 2.3 ± 0.7 mm at 6 months to 2.8 ± 0.9 mm at 24 months (*P* = 0.002). The incidence of ankylosis increased from 1.7% at 6 months to 15.0% at 24 months, while recurrent infection increased from 6.7% to 18.3% during the same period (Table 3). Fourteen teeth (11.7%) required extraction during the observation period, mainly because of severe replacement resorption and progressive mobility. These secondary clinical outcomes describe the overall clinical course after delayed replantation; however, they were not included as primary endpoints in the analysis of factors associated with root resorption.

### Time-to-event analysis of root resorption outcomes

Time-to-event analyses were performed to evaluate the timing and occurrence patterns of inflammatory and replacement resorption during the 24-month follow-up period, accounting for variable follow-up duration and censoring events (Figure S1). Kaplan–Meier analysis demonstrated distinct temporal patterns between the two types of root resorption.

Inflammatory resorption occurred predominantly during the early follow-up period, with cumulative incidence increasing mainly within the first 6 months after replantation. In contrast, replacement resorption developed progressively later, with an increasing cumulative incidence after 12 months and continued progression throughout the observation period (Figure S1).

Cox proportional hazards regression analysis was performed to identify factors associated with the timing of inflammatory and replacement resorption (Supplementary Table 4). Immature root development showed a stronger association with earlier occurrence of inflammatory resorption, whereas prolonged extra-alveolar time, dry storage, absence of root canal treatment, and delayed endodontic intervention were associated with higher hazard estimates for replacement resorption.

Because tooth extraction could prevent further observation of resorption progression, Fine–Gray competing-risk regression analysis was additionally performed with extraction considered as a competing event. The results of the competing-risk analysis for replacement resorption are presented in Supplementary Table S3.

Overall, time-to-event analysis demonstrated that inflammatory resorption was predominantly an early complication, whereas replacement resorption represented the predominant late resorption pattern following delayed replantation.

### Factors associated with root resorption after delayed replantation

Univariable and multivariable logistic regression analyses were performed to identify clinical, tooth-related, and treatment-related factors associated with root resorption during the 24-month follow-up period (Table 5).

**Table 5:** Logistic regression analysis of risk factors for root resorption after delayed replantation.

Variable	Univariate OR (95% CI)	*P*	Multivariate OR (95% CI)	*P*
**Age (years)**	0.96 (0.90–1.02)	0.18	–	–
**Male sex**	1.23 (0.65–2.34)	0.52	–	–
**Maxillary tooth (vs. mandibular)**	1.48 (0.74–2.97)	0.27	–	–
**Immature apex (open root)**	2.13 (1.05–4.35)	0.04*	1.78 (0.83–3.81)	0.14
**Extra-oral time > 2 h**	4.85 (2.01–11.70)	**< 0.001** *	3.92 (1.41–10.86)	**0.009** *
**Dry storage (vs. moist medium)**	3.67 (1.65–8.17)	**0.002** *	2.95 (1.12–7.79)	0.03*
**Rigid splinting (vs. flexible)**	1.92 (0.91–4.06)	0.09	2.04 (0.85–4.92)	0.11
**Splint duration > 14 days**	1.65 (0.79–3.44)	0.18	–	–
**No root canal treatment**	5.24 (2.24–12.25)	**< 0.001** *	4.08 (1.56–10.68)	**0.004** *
**Delayed RCT (> 14 days)**	2.86 (1.12–7.32)	0.03*	2.47 (1.01–6.02)	0.048*
**Antibiotic use (yes)**	0.87 (0.32–2.38)	0.79	–	–
**Follow-up time (months)**	1.02 (0.95–1.08)	0.56	–	–

OR: odds ratio; CI: confidence interval; RCT: root canal treatment.

Note: Univariate and multivariable logistic regression analyses were performed to evaluate associations between clinical variables and root resorption occurrence. ORs are presented with 95% CIs. The multivariable model was adjusted for clinically relevant variables and variables with *P* < 0.10 in univariate analysis. ORs >1 indicate higher odds of root resorption occurrence.

* Bold values indicate P < 0.01. P < 0.05 indicates statistical significance. Hosmer–Lemeshow goodness-of-fit test: *P* = 0.62. Model discrimination: AUC = 0.81 (95% CI: 0.74–0.88).

In univariable analysis, immature root development was associated with a higher likelihood of root resorption compared with mature roots (OR 2.13, 95% CI 1.05–4.35, *P* = 0.04). Treatment-related factors showed stronger associations, including prolonged extra-alveolar time (> 2 h; OR 4.85, 95% CI 2.01–11.70, *P* < 0.001), dry storage before replantation (OR 3.67, 95% CI 1.65–8.17, *P* = 0.002), absence of root canal treatment (OR 5.24, 95% CI 2.24–12.25, *P* < 0.001), and delayed root canal treatment (> 14 days; OR 2.86, 95% CI 1.12–7.32, *P* = 0.03). Other variables, including age, sex, tooth type, splint duration, antibiotic use, and follow-up duration, were not significantly associated with root resorption (all *P* > 0.10).

After adjustment for clinically relevant variables and factors identified in univariable analysis, several treatment-related factors remained independently associated with root resorption (Table 5). Prolonged extra-alveolar time (> 2 h) was significantly associated with increased odds of root resorption (adjusted OR 3.92, 95% CI 1.41–10.86, *P* = 0.009). Dry storage before replantation (adjusted OR 2.95, 95% CI 1.12–7.79, *P* = 0.03) and absence of root canal treatment (adjusted OR 4.08, 95% CI 1.56–10.68, *P* = 0.004) also remained significant predictors. Delayed root canal treatment showed a borderline association after adjustment (adjusted OR 2.47, 95% CI 1.01–6.02, *P* = 0.048).

**Figure 4:**
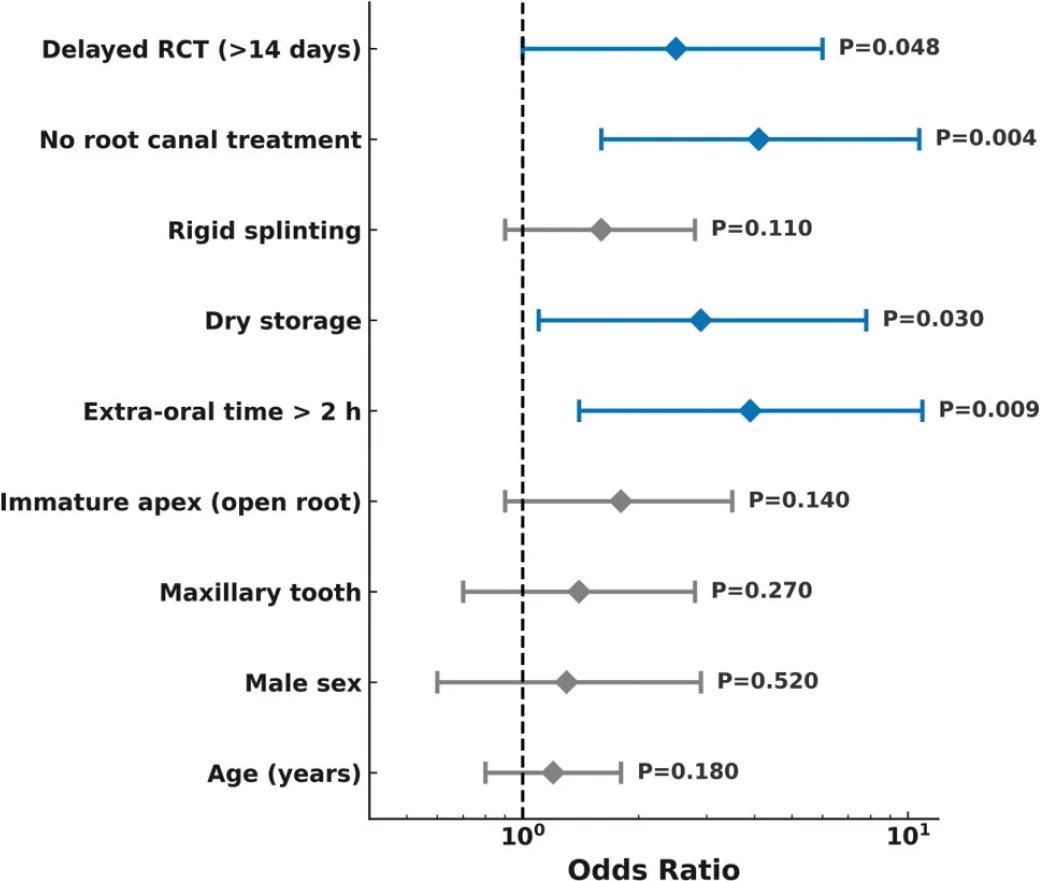
Forest plot of independent risk factors for root resorption after delayed replantation. Each horizontal line represents the 95% confidence interval (CI) for the odds ratio (OR) of each variable. Adjusted ORs and 95% CIs were obtained from the multivariable logistic regression model. Blue markers indicate variables that remained statistically significant in the multivariate model, whereas grey markers represent non-significant variables. The vertical dashed line denotes the null value (OR = 1).

The adjusted odds ratios and 95% CIs from the final multivariable regression model are presented in Figure 4. The model demonstrated that prolonged extra-alveolar time, unfavorable storage conditions, and lack of root canal treatment were independently associated with increased occurrence of root resorption after delayed replantation.

## Discussion

In this real-world cohort of delayed replantation of permanent teeth, we investigated the incidence, temporal characteristics, and clinical factors associated with root resorption during a 24-month follow-up period. The overall incidence of post-replantation root resorption was 60%, indicating that root resorption remains a frequent complication following delayed replantation. This finding is consistent with previous evidence demonstrating the vulnerability of periodontal ligament tissues after prolonged extra-alveolar exposure and the increased risk of unfavorable healing outcomes after avulsion injuries [17, 18].

Our findings demonstrated that radiographically detectable resorption-related changes developed progressively during follow-up after delayed replantation. Early superficial root surface alterations were observed during the initial months after replantation; however, these findings should be interpreted cautiously because radiographic changes cannot be directly equated with histologically confirmed microscopic surface resorption. True surface resorption may represent a transient and repairable biological process that requires histological evaluation for definitive confirmation. Therefore, in the present study, these observations were considered radiographic features compatible with superficial root changes rather than confirmed histological diagnoses. Inflammatory and replacement resorption were identified according to predefined clinical and radiographic criteria and demonstrated different temporal patterns. Inflammatory resorption was mainly observed during the early follow-up period, whereas replacement resorption became increasingly evident during later follow-up. Previous clinical and experimental studies have demonstrated that the progression and distribution of these resorption patterns may vary according to patient characteristics, treatment approaches, and follow-up duration [18, 19]. Our findings are generally consistent with previous reports and provide additional clinical information regarding the timing of detectable resorption events following delayed replantation. Previous studies have established the critical role of extra-alveolar time and storage conditions in determining periodontal ligament cell survival after avulsion [20, 21]. Andreasen and colleagues reported that when dry time exceeds 60 min, periodontal ligament cells undergo irreversible necrosis, increasing the risk of ankylosis and replacement resorption [22]. In the present cohort, prolonged extra-alveolar time and dry storage were among the variables most strongly associated with root resorption after adjustment for measured covariates. These findings support previous evidence regarding the importance of periodontal ligament preservation; however, because of the observational design, these factors should be interpreted as associated variables rather than direct causal factors. The relationship between root maturity and resorption patterns was also observed in this cohort. Teeth with immature roots demonstrated a higher frequency of inflammatory resorption, whereas mature teeth showed a greater tendency toward replacement resorption. These differences may reflect variations in periodontal healing potential, root developmental status, and pulpal conditions. However, the present study evaluated clinical associations rather than underlying biological mechanisms, and further investigations are required to confirm the mechanisms responsible for these differences [23–25].

The timing of root canal treatment represents an important clinical factor associated with outcomes following delayed replantation. In the present study, delayed or absent endodontic treatment was associated with a higher occurrence of unfavorable outcomes, particularly root resorption. The absence of root canal treatment remained independently associated with increased odds of root resorption after multivariable adjustment, while delayed endodontic intervention showed a borderline association. This association may reflect the role of endodontic management in controlling pulpal infection and reducing inflammatory responses around the root surface. However, because of the retrospective design, the present findings cannot determine whether delayed treatment directly contributed to root resorption. Treatment timing may have been influenced by clinical circumstances, injury severity, emergency management conditions, or other factors that were not fully captured in the available records.

Previous studies have emphasized the importance of timely endodontic intervention after avulsion, particularly in mature teeth where spontaneous pulp revascularization is unlikely [26, 27]. Our findings are consistent with this clinical concept, demonstrating that teeth receiving earlier endodontic management showed more favorable observed outcomes compared with teeth receiving delayed or absent treatment. Nevertheless, these findings should be interpreted cautiously because treatment allocation was not randomized, and patients requiring delayed treatment may have differed systematically from those receiving earlier intervention. Furthermore, replacement resorption is strongly influenced by the extent of periodontal ligament injury sustained at the time of avulsion, and therefore endodontic treatment alone is unlikely to completely eliminate the risk of this complication. The observed differences between inflammatory and replacement resorption support the concept that these complications represent clinically distinct healing outcomes. Inflammatory resorption is generally associated with inflammatory changes involving the root surface and surrounding tissues, whereas replacement resorption reflects progressive replacement of root structure by bone following loss of periodontal ligament function [26, 27]. Accordingly, the different temporal patterns observed in this study may reflect differences in the underlying healing responses after periodontal ligament injury. However, the present clinical data cannot directly confirm the biological mechanisms responsible for these patterns. Therefore, management strategies should consider both infection control through appropriate endodontic treatment and preservation of periodontal healing potential following replantation. Soft tissue changes, including partial root denudation associated with loss of epithelial attachment, were documented during follow-up, and topical fluoride applications were recorded in cases of delayed replacement resorption. Although these observations provide additional descriptive information regarding post-replantation management, their relationship with long-term outcomes could not be determined in this study. These findings highlight that delayed replantation outcomes represent a dynamic process influenced by multiple treatment-related and tooth-related factors. While extra-alveolar conditions appear to influence early periodontal healing responses, subsequent clinical management may also contribute to the observed clinical course. However, prospective studies are required to determine the extent to which specific interventions modify long-term outcomes.

The decline in tooth survival and functional retention observed during the 24-month follow-up period reflects the cumulative effects of root resorption, ankylosis, and secondary complications following delayed replantation. Despite these challenges, a substantial proportion of replanted teeth remained functional during the early and intermediate follow-up periods, supporting the potential clinical value of delayed replantation in selected cases. These findings are consistent with previous reports suggesting that delayed replantation may provide meaningful benefits, including preservation of esthetics, maintenance of alveolar bone contour, and temporary functional restoration, even when long-term prognosis remains uncertain [28–30].

The present findings also contribute to the ongoing clinical discussion regarding whether replantation should be considered when extra-oral dry time exceeds 60 min. Traditional recommendations have emphasized the increased risk of ankylosis and replacement resorption under these conditions. However, our results suggest that delayed replantation may still represent a treatment option in selected clinical circumstances, particularly in young patients when immediate alternatives are limited. This interpretation should not be considered evidence that delayed replantation provides outcomes equivalent to immediate replantation, but rather that preservation of the natural tooth may offer short- to medium-term functional and developmental advantages in certain situations. Previous studies have suggested that delayed replantation may contribute to preservation of alveolar bone volume and maintenance of esthetics during growth periods despite a higher incidence of late complications [31–34]. In the present cohort, survival rates exceeding 75% at 2 years indicate that some replanted teeth remained functional during the observation period; however, these findings should be interpreted within the context of the increased risk of progressive root resorption and eventual tooth loss. The real-world nature of this cohort complements previous controlled investigations by reflecting the variability encountered in routine dental trauma management.

The occurrence of tooth extraction during follow-up further emphasizes the importance of long-term monitoring after delayed replantation. In our cohort, 14 teeth (11.7%) were extracted during the 24-month observation period, mainly because of severe replacement resorption and progressive mobility. Although delayed replantation may preserve tooth function for a period of time, some teeth remain at risk for progressive deterioration. Careful follow-up is therefore required, particularly for teeth showing progressive replacement resorption or increasing mobility. Adjunctive approaches, such as decoronation or other treatment strategies, may be considered in selected cases to preserve alveolar bone volume and optimize future treatment options when long-term tooth retention becomes unfavorable.

CBCT imaging was available for a subset of patients and provided additional qualitative information regarding alveolar bone changes following replacement resorption. Quantitative Hounsfield unit measurements were not systematically collected; however, radiographic evaluation suggested that alveolar bone volume was generally preserved despite progressive replacement resorption in most cases. Mild localized cortical thinning was observed in some teeth with advanced resorption, but no extensive bone loss affecting future implant-related treatment planning was identified. Because CBCT evaluation was not performed systematically in all patients, these observations should be considered descriptive rather than definitive evidence of preserved alveolar bone quality. Future studies incorporating standardized CBCT evaluation and quantitative measurements are needed to further clarify the relationship between delayed replantation, replacement resorption, and alveolar bone preservation.

CBCT imaging was available for a subset of patients and provided additional qualitative information regarding alveolar bone changes following replacement resorption. Quantitative Hounsfield unit measurements were not systematically collected; however, radiographic evaluation suggested that alveolar bone volume was generally preserved despite progressive replacement resorption in most cases. Mild localized cortical thinning was observed in some teeth with advanced resorption, but no extensive bone loss affecting future implant-related treatment planning was identified. Because CBCT evaluation was not performed systematically in all patients, these observations should be considered descriptive rather than definitive evidence of preserved alveolar bone quality. Future studies incorporating standardized CBCT evaluation and quantitative measurements are needed to further clarify the relationship between delayed replantation, replacement resorption, and alveolar bone preservation.

Second, the absence of histological assessment limits the ability to definitively distinguish microscopic resorptive processes from radiographically detectable changes. Clinical and radiographic evaluation may not completely differentiate overlapping resorption patterns, particularly in the early stages of disease progression. Early superficial root surface changes require cautious interpretation because true surface resorption is primarily a microscopic process that may be transient and potentially repairable. As this study relied on clinical and radiographic follow-up rather than histological examination, early radiographic alterations should be considered findings compatible with superficial root changes rather than confirmed histological resorption.

Third, although examiner calibration and blinded radiographic assessment were performed to improve measurement reliability, conventional radiographic evaluation may not detect subtle early structural changes below the resolution threshold of clinical imaging techniques. CBCT imaging was performed only when clinically indicated rather than systematically, which may have resulted in underestimation of subtle changes involving the root surface or surrounding alveolar bone. Fourth, the 24-month follow-up period allowed evaluation of early and intermediate complications following delayed replantation; however, it may not fully capture long-term outcomes, particularly progressive replacement resorption, ankylosis-related changes, and eventual tooth loss. Longer follow-up periods are required to better characterize the long-term prognosis of delayed replanted teeth. Fifth, variations in adherence to IADT recommendations and differences in endodontic treatment timing reflect the real-world nature of this cohort but may have introduced treatment-related confounding. Although these variations provide valuable information regarding routine clinical practice, they limit the ability to determine whether specific management strategies independently modify long-term outcomes. In addition, selecting a single tooth per patient based on the longest extra-alveolar time or the most complete documentation may have introduced selection bias and may reduce generalizability to patients with multiple avulsed teeth or different injury patterns. The study was conducted at a single tertiary dental trauma center, which may limit generalizability to other clinical settings with different patient populations, emergency care systems, or treatment protocols. Although sensitivity analyses suggested that the main estimates were relatively stable, these analyses cannot completely eliminate potential bias associated with retrospective data collection, treatment selection, or unmeasured confounding. Future multicenter prospective studies with standardized treatment protocols and longer follow-up are needed to confirm these findings and better define causal relationships between clinical management factors and root resorption outcomes.

Despite these limitations, this study has several strengths. The inclusion of consecutive cases from a tertiary dental trauma center provides clinically relevant evidence from routine practice rather than highly controlled experimental conditions. The real-world design captures the variability in emergency management and treatment timing commonly encountered after traumatic dental avulsion. The use of standardized clinical and radiographic follow-up procedures, predefined outcome criteria, and independent examiner assessment strengthened the reliability of outcome evaluation. Furthermore, the comprehensive evaluation of patient-, tooth-, trauma-, and treatment-related variables allowed assessment of multiple clinically relevant factors associated with root resorption. The longitudinal follow-up and application of time-to-event and competing-risk analyses provided additional information regarding the timing and development patterns of resorption. Although prospective multicenter studies are required to establish causal relationships, this study provides clinically relevant evidence regarding factors associated with root resorption following delayed replantation of permanent teeth.

## Conclusion

In summary, this retrospective cohort study found that root resorption was a frequent complication following delayed replantation of permanent teeth, occurring in 60% of delayed replanted teeth during follow-up. Prolonged extra-alveolar time, dry storage, and delayed or absent root canal treatment were independently associated with a higher occurrence of root resorption after adjustment for measured covariates. These findings highlight the importance of timely management and appropriate endodontic intervention after delayed replantation. However, the retrospective single-center design, potential residual confounding, and lack of histological confirmation limit causal interpretation. Further prospective multicenter studies with longer follow-up are required to validate these associations and clarify optimal management strategies.

## Supplementary Material


