# Supplementary material for: Incidence and predictors of root resorption after delayed replantation of permanent teeth
Source: Acta Odontol Scand. 2026 Sep 23;85:46816. doi: 10.2340/aos.v85.46816 (PMC13615479; doi:10.2340/aos.v85.46816)
Supplement: Supplementary file 1 [file AOS-85-46816-s1.pdf]

Supplementary material has been published as submitted. It has not been copyedited or typeset by Acta Odontologica Scandinavica.

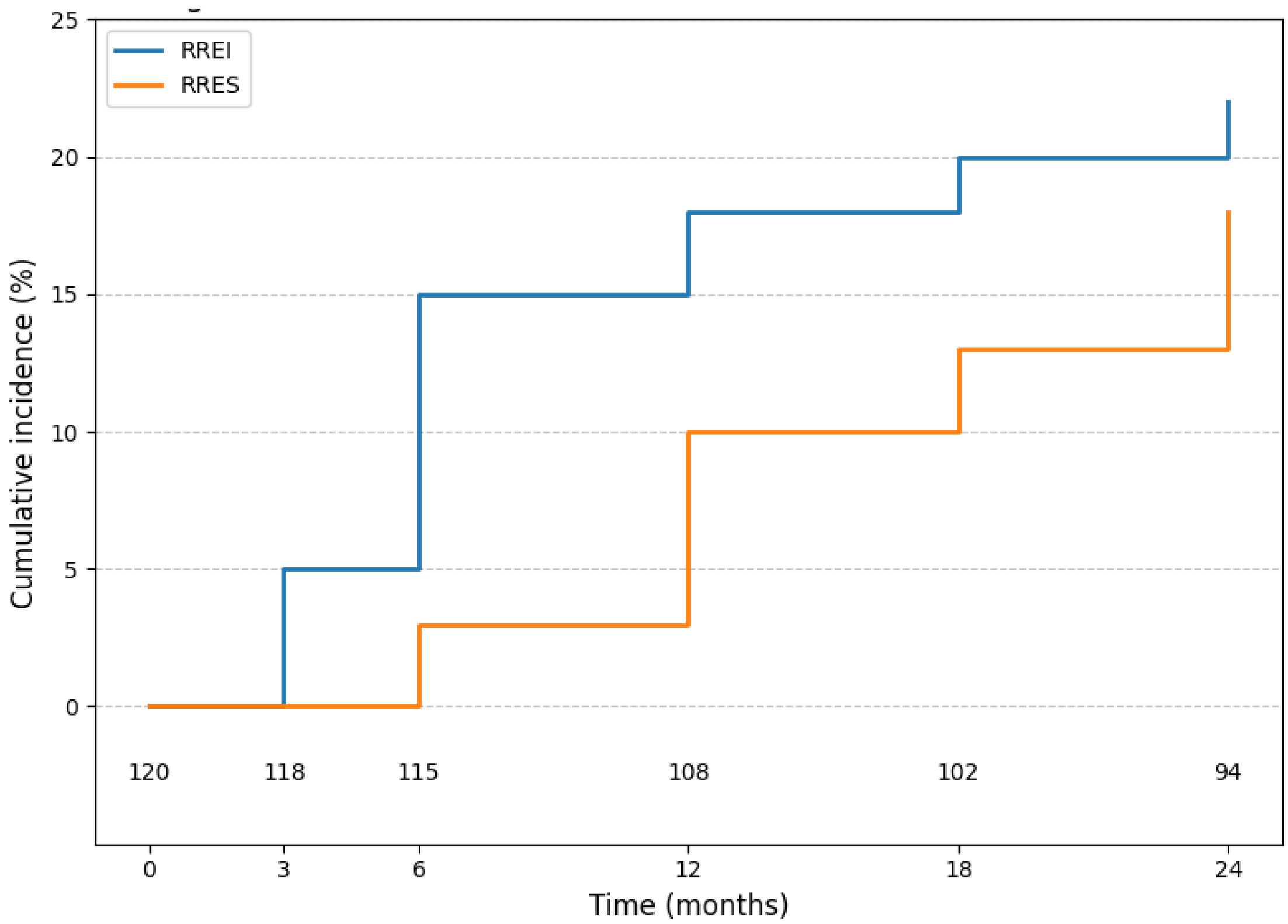

Figure S1. Cumulative incidence of root resorption following delayed replantation over 24 months.

Kaplan–Meier curves showing the cumulative incidence of inflammatory root resorption (RREI, blue line) and replacement root resorption (RRES, orange line). Teeth lost to follow-up or extracted without resorption were treated as censored events. Numbers below the x-axis indicate the teeth at risk at each time point.

Supplementary Table S1. Definition and Categorization of Variables Included in Statistical Analyses

Variable	Data source	Definition	Statistical categorization	Reference/support
Age	Clinical records	Age at the time of dental trauma	Continuous variable; categorized as ≤15 years vs >15 years for subgroup analysis	Based on developmental stage and previous dental trauma studies
Sex	Clinical records	Patient sex	Male vs female	—
Extra-alveolar time	Trauma records	Time interval from avulsion to tooth replantation	Continuous variable; categorized as ≤2 hours vs >2 hours for regression analysis	IADT guidelines and previous avulsion studies
Dry storage	Trauma records/clinical records	Period during which the avulsed tooth remained without moisture storage before replantation	Present vs absent	IADT guidelines
Storage medium	Trauma records/clinical records	Storage condition before replantation	Physiologic media (milk/saline/compatible solutions) vs non-physiologic conditions (dry storage/water)	IADT guidelines
Root maturity	Radiographic evaluation	Apical development status at the time of injury	Open apex (immature root) vs closed apex (mature root)	IADT guidelines and established dental trauma criteria
Tooth type	Clinical records/radiographs	Anatomical location of replanted tooth	Maxillary central incisor vs other teeth	Previous epidemiological studies of avulsion
Root canal treatment timing	Treatment records	Interval from replantation to initiation of root canal treatment	Continuous variable; ≤14 days vs >14 days; additional analysis >4 weeks	IADT guidelines and previous clinical studies
Splint type	Treatment records	Type of stabilization used after replantation	Flexible splint vs rigid splint	IADT guidelines
Splint duration	Treatment records	Number of days the tooth remained splinted	Continuous variable	IADT guidelines
Antibiotic administration	Medical records	Systemic antibiotic prescription after replantation	Yes vs no	IADT guidelines
Root resorption	Clinical and radiographic follow-	Occurrence of inflammatory or	Binary outcome (presence/absence);	IADT guidelines and published criteria

	up	replacement resorption based on predefined criteria	analyzed separately by resorption type	
Tooth survival	Clinical follow-up records	Retention of replanted tooth during follow-up	Survived vs extracted	Clinical outcome measure
Functional retention	Clinical examination	Asymptomatic tooth maintained in functional occlusion	Yes vs no	Clinical outcome measure
Ankylosis	Clinical and radiographic evaluation	Loss of periodontal ligament space with clinical signs of ankylosis	Present vs absent	IADT guidelines

Supplementary Table S2. Definition of study outcomes and corresponding statistical analyses

Outcome	Definition	Statistical analysis	Variables included in model
Overall root resorption (primary outcome)	Presence or absence of any radiographically or clinically detected root resorption during the 24-month follow-up period.	Univariate and multivariable logistic regression analysis.	Age, sex, root maturity, tooth type, extra-alveolar time, storage condition, root canal treatment status, root canal treatment timing, splinting type, and initial tooth mobility.
Time to inflammatory resorption	Interval from replantation to the first documented clinical and/or radiographic evidence of inflammatory resorption.	Kaplan–Meier analysis and Cox proportional hazards regression.	Age, sex, root maturity, tooth type, extra-alveolar time, dry storage, physiological storage medium, root canal treatment status, root canal treatment timing, and initial mobility.
Time to replacement resorption	Interval from replantation to the first documented clinical and/or radiographic evidence of replacement resorption.	Kaplan–Meier analysis and Cox proportional hazards regression.	Age, sex, root maturity, tooth type, extra-alveolar time, dry storage, physiological storage medium, root canal treatment status, root canal treatment timing, splinting type, and initial mobility.
Replacement resorption considering extraction as a competing event	Time to replacement resorption when tooth extraction occurred before documentation of the resorption event.	Fine–Gray competing-risk regression analysis.	Age, sex, root maturity, tooth type, extra-alveolar time, storage condition, root canal treatment status, root canal treatment timing, and initial mobility.

Tooth survival	Time from replantation to extraction of the replanted tooth.	Kaplan–Meier survival analysis.	Root resorption status, replacement resorption, inflammatory resorption, and treatment-related variables.
Functional retention	Maintenance of an asymptomatic replanted tooth in functional occlusion without pain, sinus tract formation, or clinically significant mobility.	Descriptive analysis and longitudinal comparison during follow-up.	Follow-up time, resorption status, ankylosis, infection-related complications, and clinical management variables.

Abbreviations: RCT, root canal treatment. Variables included in each model were selected based on clinical relevance and previous literature.

Supplementary Table S3. Fine–Gray competing-risk regression analysis for replacement resorption considering tooth extraction as a competing event

Variable	Adjusted sHR	95% CI	P value
Extra-alveolar time >2 hours	2.85	1.32–6.14	0.008
Dry storage before replantation	2.41	1.12–5.20	0.025
Absence of root canal treatment	3.12	1.41–6.91	0.005
Delayed RCT >14 days	1.98	0.98–4.02	0.057
Delayed RCT >4 weeks	2.36	1.08–5.17	0.032
Mature root (closed apex)	1.89	0.91–3.93	0.087
Maxillary central incisor	1.21	0.62–2.36	0.575
Initial mobility ≥ grade II	1.76	0.79–3.91	0.165
